# The Mediating Effect of the Perceived Professional Benefit of New Nurses in Cancer Hospitals on the Nursing Work Environment, Psychological Resilience, and Transition Shock: A Cross-Sectional Questionnaire Survey

**DOI:** 10.1155/2023/5741160

**Published:** 2023-08-10

**Authors:** Lu Liu, Zhuoheng Lv, Ye Zhou, Man Liu, Yan Liu

**Affiliations:** ^1^National Cancer Center, National Clinical Research Center for Cancer (NCRCC), Thoracic Surgery, Cancer Hospital, Chinese Academy of Medical Sciences & Peking Union Medical College, Beijing, China; ^2^National Cancer Center, National Clinical Research Center for Cancer (NCRCC), Intensive Care Units, Cancer Hospital, Chinese Academy of Medical Sciences & Peking Union Medical College, Beijing, China

## Abstract

**Aim:**

The aim of this study was to determine whether the relationship among the nursing work environment, psychological resilience, and transition shock was mediated by nurses' perceived professional benefits and to explore the associations among these variables.

**Background:**

Nurses' transition shock is an important factor in reducing the nursing staff turnover rate. Thus, clarifying the factors influencing nurses' transition shock has become a priority.

**Methods:**

Cross-sectional research was used in this study. A total of 200 newly graduated Chinese nurses were recruited by convenience sampling in 2022 from three tertiary hospitals in Beijing, Tianjin, and Hebei. Data were collected through questionnaires and included demographic data in addition to the perceived professional benefit scale of nurses, the nursing work environment scale, the brief resilience scale, and the transition shock scale. The data were analysed using SPSS 25.0 and the SPSS PROCESS macro programme, Model 6.

**Results:**

The perceived professional benefit of new nurses, the nursing work environment, and psychological resilience directly influenced transition shock (*p* < 0.01). The perceived professional benefit of new nurses mediated the relationship among the nursing work environment, psychological resilience, and transition shock (*p* < 0.01). The final model's mediating influence contributed 21.53% and 6.85% to the total influence.

**Conclusion:**

Nursing managers can improve nurses' perceptions of professional benefits from psychological resilience and the nursing work environment to reduce the impact of transition from school to work for new nurses. *Implications for Nursing Management*. This study provides a reference for the development of intervention strategies and training programmes to assist new nurses in cancer hospitals in effectively navigating the transition into their careers. In the future, appropriate training methods should be used at the individual cognitive, psychological, and organizational levels to improve the physical and mental health of new nurses and their ability to provide high-quality patient care.

## 1. Background

According to the World Health Organization's State of World Care report issued in 2020, the number of caregivers worldwide increased by 4.7 million between 2013 and 2018; however, there was still a shortage of 5.9 million nursing staff [1]. In China, there were 4.1 million registered nurses in 2018, yet there were only 2.7 nurses for every 1,000 residents. In 2021, the number of registered nurses in China was 5.018 million according to the Statistical Bulletin of China's Health Care Development 2021 [2]. Although an annual increase was observed, the World Health Report showed a discrepancy between the number of nurses in Europe and the United States [3]. However, the pressures experienced by nursing staff have led to increased loss of nursing professionals and high nursing staff turnover, and these setbacks have hindered the development of nursing [4]. The recruitment and retention of nursing staff are an internationally recognized priority [5]. As a new force in the health care system, newly graduated nurses play a vital role in the development of nursing quality. However, the soaring attrition rates of newly graduated nurses have resulted in the waste of educational resources and an increase in employment costs, which may have an adverse impact on initiative and instability of the residual nursing staff [6].

Nurses who graduated from school to clinical work in less than a year are defined as new nurses [7]. Due to challenges such as the gap between theory and practice, high workloads, complex relationships, and a lack of nursing skills, the process of recruiting new nurses involves “transition shock” [8]. According to studies, transition shock not only has an impact on the physical and mental health of new nurses but also increases the turnover rate, accelerates the lack of nurses' human resources, and ultimately reduces patient safety and quality of care [9, 10]. Because of transition shock, the turnover rate of new foreign nurses in the first year of their careers due to maladjustment reached 4%-5% [11]. In China, due to the impact of transition shock produced by various challenges that new nurses experience, many nurses may choose to withdraw from the nursing profession [6]; Alternately, these challenges can cause their work engagement and performance to deteriorate, which may directly affect patient safety [12].

Psychological resilience refers to inner strength, optimism, and the ability to cope effectively with adversity and to some extent reflects perseverance, resilience, and adaptability in the face of stress. Studies have shown that psychological resilience is negatively correlated with transition shock [13]. Nurses with good psychological resilience can adjust their mindset when faced with negative emotions, setbacks, difficulties, and adversities and restore their enthusiasm and attitude towards nursing work, which is conducive to team building and career development [14].

The nursing work environment refers to the organizational characteristics that facilitate or limit the professional practice of nursing, including the relationship between nurses and administrators, with physicians, and the status of nursing staff in the hospital hierarchy [15]. Previous studies have shown that an optimal nursing work environment is not only effective in improving nurse outcomes but also in increasing job satisfaction and a sense of professional and organizational identity and job adaptability, reducing the impact of transition shock [16, 17]. New nurses are at the beginning of their careers, and the nursing work environment is critical to their growth during this period.

A sense of professional benefit refers to the emotional state in which individuals perceive the rewards and benefits brought by their occupations and agree that their current occupations can promote their overall growth [18]. Related studies have shown that new nurses experience increased stress. When they change from students to nurses, they face a dramatic change in role and environment and experience feelings of confusion, doubt, bewilderment, and unclear orientation. Their perceived sense of career benefit will influence their career choice or career development and reduce the pressure and confusion caused by the impact of transition shock [19, 20].

However, since China's standardized system for training new nurses is still in the exploratory stage, training programmes in cancer hospitals frequently place a greater emphasis on theoretical knowledge and clinical practice than on work pressure and the psychological adjustment that accompanies a change in role for new nurses [21]. Faced with cancer patients and their families, new nurses in cancer hospitals feel greater physical and mental pressure and are more likely to experience job burnout and turnover intention [22]. Previous studies have concentrated on general hospitals, while the group of new nurses in cancer hospitals has received less attention [23].

With the increasing incidence and mortality rate of tumours, the demand for high-quality cancer care services is increasing. Currently, new nurses in cancer hospitals deal with a shortage of nursing human resources, demanding job responsibilities, and a variety of stressors associated with the increasing complexity of cancer care, making the transition challenging [24]. Nurses working at cancer hospitals deal with special patients who require prolonged medical care, have numerous complications, and endure significant economic pressure. The high expectations of patients and their families for medical staff places more pressure on nurses in cancer hospitals than those in general hospitals [25].

The transition shock model framework was used as a guide for this study. The model covered three aspects: the source of transformation shock (role, interpersonal relationship, responsibility, knowledge, and skills), the feelings (confusion and doubt), and the level of influence (physiology, emotion, social culture, and development thought) [26]. The theory states that the occurrence of transition shock is affected by several factors. According to research [27–29], improving external factors, such as strengthening clinical teachers' teaching practices, creating a positive working environment, and providing timely support to new nurses, can reduce the impact of transformation. Internal factors such as positive coping styles and self-efficacy are negatively correlated with transition shock, and the relationship between resilience and transition shock is elusive [30–32].

Therefore, this study verified the path mechanism by which the external environment and internal psychological resilience affect the transition shock of new nurses in cancer hospitals and explored the partial mediating role of nurses' perceived professional benefit in managing the nursing work environment, coping with psychological resilience, and experiencing transformation shock. Based on the hypotheses proposed in this framework and previous studies, we formulated the following hypotheses in our study: (1) the nursing work environment (external factors) and psychological resilience (psychological factors) directly influence the impact of transition shock; (2) nurses' perceived professional benefits (personal factors) influence transition shock; and (3) the nursing work environment and psychological resilience affect transition shock through nurses' perceived professional benefit.

Surprisingly, few empirical studies have investigated the impact of nurses' perceived professional benefits on transition shock, including the mediating effects of nurses' perceived professional benefits, particularly in China. However, understanding this link is critical for designing effective ways to minimize turnover intention.

The major goal of our research was to examine the link between transition shock and personal cognitive factors, psychological factors, and environmental factors in new nurses in cancer hospitals. This knowledge can help nursing managers implement effective interventions to reduce transition shock and turnover intentions among new nurses, reduce the loss of nursing human resources, ensure nursing safety, and improve clinical nursing quality.

## 2. Methods

### 2.1. Methodological Rigour and Quality of the Study

First, according to the purpose of the study, we chose suitable assessment tools. In addition, our study followed the STROBE checklist for reporting findings.

### 2.2. Study Design and Participants

This study adopted a cross-sectional design. The convenience sampling method was utilized. In 2022, newly registered nurses from three tertiary cancer hospitals in the Beijing, Tianjin, and Hebei regions were enrolled in this study. Employing the method for calculating the sample size applicable to continuous variables [33], we postulate a variance of 25 for nurses' perception of professional benefit. With a precision *δ*, defined as the distance from the mean to the limit, set at 3.5, a minimum sample size of 199 is deemed necessary for the study. A total of 212 new nurses working in the three participating hospitals were invited to participate in the study. After checking the questionnaires they completed and removing those with incomplete responses, 200 questionnaires were considered for data analysis. The response rate was 94.33%. The inclusion criteria were as follows: (1) acquiring a nursing qualification certificate and a nursing practice certificate, (2) <1 year of work experience, and (3) voluntary participation in this study and signed informed consent. The exclusion criteria were as follows: maternity leave, sick leave, internship, rotation, and refresher nurses. Newly graduated nurses have different abilities to withstand stress compared to experienced nurses [34], and different levels of stress may affect nurses' perceived professional benefits, transition shock, and psychological resilience [25]. To avoid interference factors, we also excluded new nurses who had worked in two or more hospitals in the past year. Ethical approval for this study was obtained from the Ethics Committee of the Cancer Hospital Chinese Academy of Medical Sciences (21/525-3196).

### 2.3. Variables

General sociodemographic information was gathered through a self-designed questionnaire and included age, sex, marital status, education level, and other information.

The nurses' perception of professional benefit was assessed with a previous questionnaire [35]. The questionnaire was used to assess the perception of the gains and benefits brought by the nursing profession and to identify whether nursing can promote the overall growth of the self. Positive career perception (5 items), family and friend recognition (7 items), team belonging (5 items), good nurse-patient relationships (6 items), and self-growth (6 items) constituted a total of 29 items. The items were rated on a 5-point scale from 1 (strongly disagree) to 5 (strongly agree). The total score was proportional to the nurses' perception of professional benefit. The total Cronbach's alpha coefficient of the scale was 0.958, and Cronbach's *α* coefficient of each dimension was between 0.821 and 0.893. In this study, the scale's reliability coefficient was 0.953.

The Chinese version of the Practice Environment Scale (PES) was compiled by Professor Lake [36]. After agreement from the original scale author, it was translated into Chinese by Wang and Li [37]. The scale included five dimensions, nurses' participation in hospital affairs (8 items), the basis of high-quality nursing services (9 items), managers' ability and leadership style (4 items), sufficient manpower and material resources (4 items), and medical cooperation (3 items), for a total of 28 items. The items were rated on a 4-point scale ranging from 1 (strongly disagree) to 4 (strongly agree) for a total score of 31–124 points. Higher scores indicated higher levels of the perceived work environment. Cronbach's *α* of the scale was 0.910.

Psychological resilience was assessed by the Brief Resilience Scale (BRS) [38]. The BRS consists of six items, three positive and three negative scored items. The items are measured on a 5-point scale, with 1 representing “does not describe me at all” and 5 representing “describes me very well.” The data collected were recorded prior to analysis since item 2 (I have a hard time making it through stressful events), item 4 (It is hard for me to snap back when something bad happens), and item 6 (I tend to take a long time to get over setbacks in my life) were reverse coded. This scale was specifically used to assess individuals' ability to maintain health when dealing with stress, especially health-related stress or stressful events. The average score of items was divided into three groups: low elasticity (1.00–2.99), medium elasticity (3.00–4.30), and high elasticity (4.31–5.00). Cronbach's *α* of the BRS was 0.71.

The transition shock scale for newly graduated nurses (TSS-NGNs) evaluates their emotional burden as they move from the school environment to clinical practice [39]. The scale includes four dimensions: shock from organizational culture and climate, shock from knowledge and skills, psychological shock, and physical shock. There are 27 items in total. The ratings are measured on a 5-point scale with 1 representing “strongly disagree” and 5 representing “strongly agree,” for a total score ranging from 27 to 135 points. A higher score indicates a higher level of transition shock. Cronbach's *α* scale for each domain ranged from 0.86 to 0.94.

### 2.4. Survey Methods

The researchers explained the background, purpose, and methods of this study to the nurses who met the inclusion criteria. Following informed consent, a 10- to 20-minute online survey was conducted via mobile phone. Participants accessed the questionnaire by scanning the questionnaire's two-dimensional code created by Questionnaire Star. To ensure that the survey data submitted were complete and did not contain missing data, questions could not be skipped.

### 2.5. Data Analysis

Descriptive statistical analysis, including mean, standard deviation, frequency, and percentage, was performed using SPSS 26.0 software. Using the SPSS PROCESS macro program, Model 6, we tested the mediating effect. Data were tested for normality using the Kolmogorov‒Smirnov (K-S) test. A nonparametric correlation method (Spearman's rho) was used to test the relationships among the nurses' perception of professional benefit, nursing work environment, psychological resilience, and transition shock. The structural equation model (SEM) was used to analyse the associations among the nursing work environment, psychological resilience, nurses' perceived professional benefit, and transition shock. The product coefficient test and bootstrap sampling methods were used to test the mediating effect. Multidimensional indicators were used to comprehensively assess the model's acceptability. Therefore, to evaluate the fit quality of the model and data, several goodness-of-fit indicators were employed: chi-square (*x*^2^), chi-square/degrees of freedom (*x*^2^/d*f*), root mean square error of approximation (RMSEA), goodness-of-fit index (GFI), standardized root mean square residual, and nonnormed fit index (NFI). The standard for good model fit was <3, RMSEA <0.1, NFI, and CFI >0.9. The statistical significance level was set at *α* = 0.01. Before proceeding with the regression analysis, we verified that our dataset satisfied the fundamental prerequisites for regression. These prerequisites encompassed the normal distribution of residuals, linearity of relationships, homoscedasticity, and the absence of multicollinearity.

## 3. Results

### 3.1. Characteristics of Participants

Table 1 presents the demographic and work-related characteristics of the participants. The effective sample size was 200. Most participants were female (82%). The average age of the participants was 21.82 (3.37) years, and 97% of the participants were single. In terms of education level, 49% of the participants held a college degree. The majority of participants were from a one-child family (72%), and 34% of participants had a monthly income of more than 4000¥. More details can be found in Table 1.

### 3.2. Descriptive Statistics of Scales

Table 2 shows the descriptive statistics of the nurses' perceived professional benefit, nursing work environment, psychological resilience, and transition shock. The mean scores on the nurses' perceived professional benefit were 46.25 (24.12), with the highest scores in the self-growth dimension (9.08 (5.16)) and the lowest scores in the family and friends' recognition dimension (7.90 (4.49)). The mean score for the nursing work environment was 95.35 (12.77). The scores of each dimension from high to low are the basis of high-quality nursing services, nurses' participation in hospital affairs, sufficient manpower, and material resources medical cooperation (31.30 (4.41); 26.67 (4.17); 13.58 (2.06); 10.65 (1.60), respectively). The mean score for psychological resilience was 17.07 (3.65). The mean score for transition shock was 51.45 (22.34). The organizational culture and climate dimensions had the highest scores (15.46 (7.02)), and the knowledge and skills dimension had the lowest score (10.41 (4.60)).

### 3.3. Correlation Test

Table 3 shows the associations among the nurses' perceived professional benefit, nursing work environment, psychological resilience, and transition shock. Nurses' perceived professional benefit was positively correlated with psychological resilience (*r* = 0.420, *p* < 0.01) and the nursing work environment (*r* = 0.103, *p* < 0.01). Nurses' perceived professional benefit was negatively related to transition shock (*r* = −0.421, *p* < 0.01). The nursing work environment was positively correlated with psychological resilience (*r* = 0.103, *p* < 0.01) and was negatively related to transition shock (*r* = −0.437, *p* < 0.01). Psychological resilience was negatively related to transition shock (*r* = −0.517, *p* < 0.01).

### 3.4. The Mediating Role of Nurses' Perceived Professional Benefit on the Relationships of Nursing Work Environment, Psychological Resilience, and Transition Shock

Prior to assessing the mediation effect, the assumptions of regression analysis were scrutinized. The Durbin–Watson statistic emerged at 1.321, in close proximity to the ideal value of 2, thus suggesting the absence of autocorrelation among residuals. The maximum variance inflation factor was identified as 1.249, less than the critical threshold of 10, further indicating that multicollinearity was not a concern. In addition, the analysis of residuals affirmed the model's linearity and confirmed both the normality and homoscedasticity of errors. Harman's single-factor test was employed to perform an exploratory factor analysis on all measured items associated with the nursing work environment, nurses' perceived professional benefit, psychological resilience, and transition shock, without any rotation. The analysis revealed three common factors with an eigenvalue exceeding 1. The first common factor accounted for 41.55% of the total variance, falling below the 50% threshold. This finding suggests that the research data were not subjected to common method bias.

**Figure 1:**
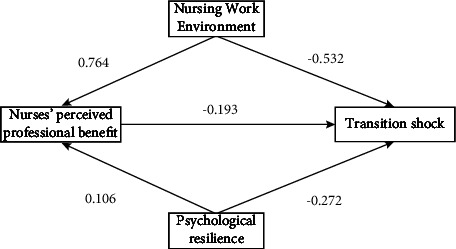
The mediating role of nurses' perceived professional benefit.

As depicted in Figure 1, we applied the Sobel stepwise method to estimate the mediation effect size. The term *α* symbolizes the intensity of the association between the independent variable and the mediator. In this instance, based on univariate regression analysis, both the nursing work environment and psychological resilience were found to enhance nurses' perceived professional benefit, with *α*1 = 0.764 (*p* < 0.001) and *α*2 = 0.106 (*p* < 0.001), respectively. The term *β* designates the strength of the relationship between the mediator and the dependent variable, while accounting for the influence of the independent variable. In our case, after regressing all variables towards transition shock, we observed a negative effect *β* = −0.193 (*p* < 0.001) from nurses' perceived professional benefit to transition shock. Finally, the *αβ* term represents the product of coefficients, essentially quantifying the variance in the dependent variable that can be attributed to the independent variable via the mechanism of the mediator.  Table 4 demonstrates that nurses' perceived professional benefit plays a partial mediating role in the relationships among nursing work environment, psychological resilience, and transition shock. The contribution rates of the mediating effect to the total effect were 21.53% and 6.85%, respectively. As illustrated in Table 4 and Figure 1, the nursing work environment had a significant direct impact on nurses' perceived professional benefit (*p* < 0.01) and a significant direct and indirect impact on transition shock (*p* < 0.01). In addition, the outcomes demonstrated that resilience significantly influenced transition shock both directly and indirectly (*p* < 0.01).

## 4. Discussion

This study concentrated on newly recruited nurses in cancer hospitals and investigated the relationships among the nursing work environment, psychological resilience, nurses' perceived professional benefit, and transition shock. Based on the understanding of transformation shock theory and previous research, a hypothetical model was constructed and the relationships among these variables were verified. The findings of this study confirmed the research hypotheses.

First, our study demonstrated a negative correlation between the nursing work environment and transition shock. This means that the better the nursing work environment is, the lower the transition shock of nurses, which is consistent with previous studies [4]. The working environment is a comprehensive system that integrates physiology, psychology, society, and specialty. It is a place where nurses work and socialize daily, which is inseparable from their behaviour [40]. A good working environment improves the job satisfaction of nurses, enables them to better integrate into their new environment, and reduces the impact of role transformation [41]. The nursing work environment includes five aspects: nurses' participation in hospital affairs, the basis of high-quality nursing service, the ability and leadership of managers, sufficient manpower and material resources, and medical cooperation. Al Sabei et al. [42] observed that having more people involved in hospital affairs can help reduce the work pressure and turnover intention of nurses. By providing new nurses with more opportunities to participate in the decision-making processes of hospital affairs through high-level organizational support and good cooperative relationships, nursing managers can strengthen nurses' sense of belonging and reduce the risk of new nurses' resignation during the transition period. Benner and Duchess's theory [8] suggests that nurses obtain significant knowledge in their initial 6 months of employment, enabling them to actively participate in clinical work. For nurses in cancer hospitals, clinical knowledge needs to be more systematic and professional. Therefore, to ensure that new nurses are capable of participating in their work, nursing managers can concentrate on developing a knowledge system 6 months prior to the admission of new nurses by developing a planned training programme. They need to also concentrate on improving nurses' knowledge capacity for clinical transformation. A good nursing work environment should meet both physical and psychological needs. According to the American psychologist Bronfenbrenner [43], the theory of human development emphasizes that supportive interactions in the microenvironment promote personal development. Therefore, clinical nursing managers need to pay attention to the psychological and emotional requirements of new nurses to create a positive working atmosphere of mutual assistance.

Second, this study demonstrated a positive correlation between the work environment of nurses and their perceived professional benefit and a negative correlation between nurses' perceived professional benefit and transition shock. Not only did the nursing work environment directly affect transition shock, but it also indirectly affected transition shock via nurses' perceived professional benefit. The nurses' perceived professional benefit played a partial mediating role between work environment and transition shock, accounting for 21.53% of the total influence. This may be due to the knowledge that a healthy work environment can provide new nurses with a sense of safety, belonging, and positivity. In turn, this allows individuals to believe that they can cope with the challenges of their environment and to develop the courage to accept new things. A good environment enhances innovation among nurses and helps them provide quality care to their patients. When nurses are assisted in implementing safe, standard, competent, and compassionate services, they tend to feel a sense of self-identity, thus enhancing their sense of professional benefit [44]. Nurses' perceived professional benefit is a positive and pleasant emotional experience that brings them satisfaction in the process of work. It is an internal incentive factor for the career development of nurses that helps them cope with work challenges and reduces the psychological impact [45]. Therefore, healthy working conditions and the environment are crucial for the mental health and work adjustment of nursing staff. A study demonstrated that job characteristics and an interpersonal atmosphere can predict job performance and satisfaction [46]. It is further suggested that nursing managers acknowledge the role that the nursing environment plays in improving nurses' sense of belonging and interpersonal skills while reducing psychological pressure in the transition period to help them better accept and complete their clinical nursing duties.

Third, our study demonstrated that nurses' perceived professional benefit also mediated the relationship between psychological resilience and transition shock, and the mediating effect accounted for 6.85% of the total influence. There was a positive correlation between psychological resilience and nurses' perceived professional benefit, and there was a negative correlation between psychological resilience and transformation impact. That is, the higher the level of psychological resilience, the more nurses feel a sense of professional benefit, and the higher the sense of professional benefit, the lower the transformation impact. Resilience is a coping mechanism that helps individuals deal with challenges and pressures [47]. Therefore, good psychological resilience can help new nurses derive a positive career perception and ease the pressure of transitioning. A study showed that effective emotional control and problem-centered coping strategies can effectively manage stress and improve resilience by providing motivating images and enhancing self-humour [48]. Another study using basic psychological needs theory [49] further explored the underlying mechanisms between resilience and positive psychological functions. The theory proposes that the best state of positive psychology depends on the satisfaction of three basic psychological needs: autonomy, competence, and relationality. Research shows that managers' autonomy support and positive feedback can mobilize the three basic psychological needs of autonomy, competence, and belonging of employees, thus increasing their job satisfaction and reducing the rate of turnover [50]. Therefore, managers can improve the psychological satisfaction of new nurses through encouragement, affirmation, and praise to help reduce psychological pressure.

The results of this study revealed that nurses' sense of professional benefit has an important impact on their transformation. Therefore, nursing managers should pay attention to and improve the work experience of new nurses and take corresponding measures to improve the sense of professional benefit to help nurses complete the transition. To improve the positive benefit to nurses, Rui et al. [51] suggested practising a mindful diet and sharing pleasant or unpleasant events that occurred during daily work, health education, and meditation. These activities ensure that nurses can calmly accept reality, which helps them improve their emotional responses and stress management abilities, experience things from a positive perspective, and improve their professional values. Dai et al. [52] use the Satir model to train nurses, which can help nurses understand restrictive beliefs and form positive professional cognition. When nurses change their communication mode and improve their relationships with others, they can improve their growth and effectively improve their professional benefit level. The professional benefits of nursing may be realized if the job is accompanied by a stable income, guaranteed work, services for relatives, and flexible working hours. Nursing managers should guide new nurses to rationally and objectively evaluate their professional characteristics from multiple perspectives and dimensions through these practical benefits.

### 4.1. Study Limitations and Future Research

Although the findings of this study support the initial research hypothesis, there are still some limitations. First, this was a cross-sectional study, which may not illuminate the causal relationship between variables. Therefore, a longitudinal study design can be performed in the future to further investigate the relationship between the variables. Second, although this study selected new nurses from three hospitals in Beijing, Tianjin, and Hebei as the research subjects, the sample size was small due to the limiting factor of new nurses solely from cancer hospitals. A large sample survey can be conducted in the future to improve the universality of the results of this study. Finally, only Chinese nurses participated in the study. Further exploration should include nurses from other countries. We will actively participate in international conferences related to nursing management, further broadening this field of study.

## 5. Conclusion

This study indicates that increasing the professional benefit of nurses by providing a healthy nursing work environment and improving psychological resilience can reduce the impact of transformation. Therefore, nursing managers should pay attention to the role of the external environment and internal psychological construction during the transition period of new nurses and encourage new nurses to participate in decision-making and nursing practice. It is also necessary to develop and design prejob training programs and intervention measures that are suitable for new nurses working in cancer hospitals and select various implementation strategies based on the actual situation to implement training programs purposefully and appropriately.

## Figures and Tables

**Table 1:** Characteristics of the study sample.

Variables	Categories	*n* (%)
Sex	Female	164 (82)
	Male	36 (18)

Age	<25	188 (94)
	≥25	12 (6)

Marital status	Married	6 (3)
	Single	194 (97)

Education level	College	98 (49)
	Associate's degree or less	8 (4)
	Undergraduate	82 (41)
	Postgraduate and more	12 (6)

From one-child family	No	144 (72)
	Yes	56 (28)

Monthly income	1000–2000¥	40 (20)
	2001–3000¥	84 (42)
	3001–4000¥	8 (4)
	>4000¥	68 (34)

Home address and work unit in the same city	No	8 (4)
	Yes	192 (96)

Employment relationship	Formal incorporation	8 (4)
	Personnel agency	8 (4)
	Contract employment	184 (92)

Were you ever a class cadre during college?	No	80 (40)
	Yes	120 (60)

**Table 2:** The level of variables (*n* = 200).

Variables	Minimum value	Maximum value	*M* (SD)
Nurses' perceived professional benefit	29	145	46.25 (24.12)
Positive career perception	5	25	8.49 (4.30)
Family and friends' recognition	5	25	7.90 (4.49)
Team belonging	6	30	9.08 (5.16)
Good nurse-patient relationships	6	30	8.99 (5.12)
Self-growth	7	35	11.79 (5.85)
Nursing work environment	36	112	95.35 (12.77)
Nurses' participation in hospital affairs	8	32	26.67 (4.17)
The basis of high-quality nursing services	9	36	31.30 (4.41)
Managers' ability and leadership style	6	16	13.35 (1.78)
Sufficient manpower and material resources	9	16	13.58 (2.06)
Medical cooperation	4	12	10.65 (1.60)
Psychological resilience	6	30	17.07 (3.65)
Transition shock	27	132	51.45 (22.34)
Physical	6	30	12.03 (6.12)
Psychological	8	40	15.46 (7.02)
Knowledge and skills	5	25	10.41 (4.46)
Organizational culture and climate	8	40	13.56 (6.82)

**Table 3:** Correlations of variables.

	1	2	3	4
(1) Psychological resilience	1			
(2) Nursing work environment	0.103 (*p* < 0.01)	1		
(3) Nurses' perceived professional benefit	0.194 (*p* < 0.01)	0.420 (*p* < 0.01)	1	
(4) Transition shock	−0.517 (*p* < 0.01)	−0.437 (*p* < 0.01)	−0.421 (*p* < 0.01)	1

(*p*) value.

**Table 4:** Mediation effect size results.

Variables	Total effect (Sobel *p*)	Direct effect (Sobel *p*)	Indirect effect (Sobel *p*)
Nursing work environment ⟶ nurses' perceived professional benefit ⟶ transition shock	−0.678 (*p* < 0.01)	−0.532 (*p* < 0.01)	−0.146 (*p* < 0.01)
Psychological resilience ⟶ nurses' perceived professional benefit ⟶ transition shock	−0.292 (*p* < 0.01)	−0.272 (*p* < 0.01)	−0.020 (*p* < 0.01)

(*p*) value.

## Data Availability

The data used to support the findings of this study are available from the corresponding author upon request.
